# Virologic and Immunologic Response to cART by HIV-1 Subtype in the CASCADE Collaboration

**DOI:** 10.1371/journal.pone.0071174

**Published:** 2013-07-30

**Authors:** Giota Touloumi, Nikos Pantazis, Marie-Laure Chaix, Heiner C. Bucher, Robert Zangerle, Anne-Marte Bakken Kran, Rodolphe Thiebaut, Bernard Masquelier, Claudia Kucherer, Antonella d'Arminio Monforte, Laurence Meyer, Kholoud Porter, for CASCADE Collaboration in EuroCoord

**Affiliations:** 1 Athens University Medical School, Athens, Greece; 2 Université Paris Descartes EA 3620, AP-HP, Laboratoire de Virologie, CHU Necker-Enfants Malades, Paris, France; 3 Basel Institute for Clinical Epidemiology and Biostatistics, Basel, Switzerland; 4 University Hospital Innsbruck, Innsbruck, Austria; 5 Oslo University Hospital Ullevål, Department of Microbiology, Oslo, Norway; 6 University Bordeaux, ISPED, INSERM, Centre INSERM U897-Epidemiologie-Biostatistique, Bordeaux, France; 7 Hospital Pellegrin, Bordeaux, France; 8 Robert Koch Institute, Berlin, Germany; 9 Institute of Infectious Diseases, San Paolo Hospital, University of Milan, Milan, Italy; 10 INSERM U 1018, AP-HP, Université Paris Sud, Paris, France; 11 MRC Clinical Trials Unit, London, United Kingdom; National Institute of Allergy and Infectious Diseases, United States of America

## Abstract

**Background:**

We aimed to compare rates of virologic response and CD4 changes after combination antiretroviral (cART) initiation in individuals infected with B and specific non-B HIV subtypes.

**Methods:**

Using CASCADE data we analyzed HIV-RNA and CD4 counts for persons infected ≥1996, ≥15 years of age. We used survival and longitudinal modeling to estimate probabilities of virologic response (confirmed HIV-RNA <500 c/ml), and failure (HIV-RNA>500 c/ml at 6 months or ≥1000 c/ml following response) and CD4 increase after cART initiation.

**Results:**

2003 (1706 B, 142 CRF02_AG, 55 A, 53 C, 47 CRF01_AE) seroconverters were included in analysis. There was no evidence of subtype effect overall for response or failure (p = 0.075 and 0.317, respectively) although there was a suggestion that those infected with subtypes CRF01_AE and A responded sooner than those with subtype B infection [HR (95% CI):1.37 (1.01–1.86) and 1.29 (0.96–1.72), respectively]. Rates of CD4 increase were similar in all subtypes except subtype A, which tended to have lower initial, but faster long-term, increases.

**Conclusions:**

Virologic and immunologic response to cART was similar across all studied subtypes but statistical power was limited by the rarity of some non-B subtypes. Current antiretroviral agents seem to have similar efficacy in subtype B and most widely encountered non-B infections in high-income countries.

## Introduction

HIV-1 is characterized by its high genetic diversity and is classified into 4 groups, M, N, O and P [1] with group M dominating the epidemic worldwide. Group M is further classified into 9 subtypes (A-D, F-H, and K) and an increasing number of inter-subtype circulating recombinant forms (CRFs) and unique recombinant forms (URFs). Globally, subtype C accounts for almost 48% of infections and dominates the epidemic in Southern Africa, India and China, followed by subtype A (≈27%) which dominates in Eastern Africa, Eastern Europe and Central Asia. Subtype B accounts for about 12% of HIV infections worldwide [2], [3] and, although it dominates in high-income countries, the prevalence of non-B subtypes has increased in those countries in recent years, mainly due to mixing of populations [2], [4].

Antiretroviral drugs have been developed mainly using subtype B as the reference virus and in vitro studies have suggested that subtype may affect susceptibility to certain drugs [2], [5]–[7]. Given the globally increasing HIV-1 genetic heterogeneity and wider availability of combination antiretroviral therapy (cART), it is important to assess whether it is equally active against all subtypes and CRFs. Although parallel epidemics of different subtypes are now commonly observed, they tend to be restricted to specific ethnic or risk groups making comparisons across subtypes difficult.

Most of the previous studies assessing virological and immunological response to cART by HIV-1 subtype had the serious limitation of grouping all non-B subtypes together due to small numbers [8]–[14]. The few studies that examined the effect of single subtypes were restricted to specific subtypes depending on the geographic region from which the study population was derived [15]–[17]. Taking advantage of CASCADE, a large international collaboration of seroconverter cohorts, we aimed to investigate the effect of specific HIV-1 subtypes on immunological and virological response to cART in persons living in high-income countries.

## Methods

### Ethics statement

All collaborating cohorts received approval from their respective or national ethics review boards. Ethics approval for CASCADE collaborating cohorts has been granted by the following committees: Austrian HIV Cohort Study: Ethik-Kommission der Medizinischen Universität Wien, Medizinische Universität Graz – Ethikkommission, Ethikkommission der Medizinischen Universität Innsbruck, Ethikkommission des Landes Oberösterreich, Ethikkommission für das Bundesland Salzburg; PHAEDRA cohort: St Vincent's Hospital, Human Research Ethics Committee; Southern Alberta Clinic Cohort: Conjoint Health Research Ethics Board of the Faculties of Medicine, Nursing and Kinesiology, University of Calgary; Aquitaine Cohort: Commission Nationale de l'Informatique et des Libertés; French Hospital Database: Commission nationale de l'informatique et des libertés CNIL; French PRIMO Cohort: Comite Consultatif de Protection des Personnes dans la Recherché Biomedicale; SEROCO Cohort: Commission Nationale de l'Informatique et des Libertés (CNIL); German HIV-1 Seroconverter Study: Charité, University Medicine Berlin; AMACS: Bioethics & Deontology Committee of Athens University Medical School and the National Organization of Medicines; Greek Haemophilia Cohort: Bioethics & Deontology Committee of Athens University Medical School and the National Organization of Medicines; ICoNA cohort: San Paolo Hospital Ethic Committee; Italian Seroconversion Study: Comitato etico dell'Istituto Superiore di Sanità; Amsterdam Cohort Studies in Homosexual Men and IDUs: Academic Medical Centre, University of Amsterdam; Oslo and Ulleval Hospital Cohorts: Regional komite for medisinsk forskningsetikk – Øst- Norge (REK 1); Badalona IDU Hospital Cohort: Comité Ético de Investigación Clínica del Hospital Universitari Germans Trias i Pujol; CoRIS-scv: Comité Ético de Investigación Clínica de La Rioja; Madrid Cohort: Ethics Committee of Universidad Miguel Hernandez de Elche; Valencia IDU Cohort: Comité Etico de Investigación Clínica del Hospital Dr. Peset-Valencia; Swiss HIV Cohort Study: Kantonale Ethikkommission, spezialisierte Unterkommission Innere Medizin, Ethikkommission beider Basel, Kantonale Ethikkommission Bern, Comité départemental d'éthique de médecine et médecine communautaire, Commission d'éthique de la recherche clinique, Université de Lausanne, Comitato etico cantonale, Ethikkommission des Kantons St.Gallen; UK Register of HIV Seroconverters: South Birmigham REC; Early Infection Cohorts: Kenya Medical Research Institute, Kenyatta National Hospital, Uganda Virus Research Institute Science and Ethics Committee, Uganda National Council for Science and Technology, Uganda Virus Research Institute Science and Ethics Committee, Uganda National Council for Science and Technology, University of Zambia Research Ethics Committee, Emory IRB, National Ethics Committee of Rwanda, University of Cape Town Research Ethics Committee, University of Kwazulu Natal Nelson R Mandela School of Medicine; Genital Shedding Study Cohort: University Hospitals of Cleveland, IRB for Human Investigation (CWRU), AIDS Research Committee (ARC), STD/AIDS Control Programme, Uganda Ministry of Health, Committee on Human Research (CHR), Office of Research Administration (UCSF), Biomedical Research & Training Institute (BRTI) – Zimbabwe, Institutional Review Office, Fred Hutchinson Cancer Research Center, Medical Research Council of Zimbabwe (MRCZ). Written informed consent was obtained from all participants.

### Study population

We used data from CASCADE (Concerted Action of Seroconversion to AIDS and Death in Europe), a collaboration of 28 cohorts of individuals with well-estimated dates of HIV seroconversion (seroconverters) [18]. Seroconversion dates were estimated by various methods, most frequently as the midpoint between the last documented negative and first positive HIV antibody test dates with an interval of less than 3 years between tests (84.6%). The remainder were estimated through the availability of laboratory evidence of acute seroconversion (PCR positivity in the absence of HIV antibodies or antigen positivity with fewer than four bands on Western blot), or as the date of seroconversion illness with both an earlier documented negative and a later positive HIV test not more than 3 years apart.

Individuals followed-up in two African cohorts in CASCADE were excluded as treatment guidelines applied to these populations differ from those in high-income countries. We also excluded individuals who seroconverted <1996 when cART became available. Eligible individuals were >15 years at seroconversion, started a stable cART regimen (i.e. at least 60 days) and had both CD4 and HIV-RNA measurements available at baseline (i.e. within the last 6 months prior to cART initiation) and while on their first cART regimen. As the main focus of this analyses was on response to cART, individuals with HIV-RNA<500 copies/ml at cART initiation were also excluded.

Follow-up time was censored at the first major treatment modification (i.e. change of cornerstone drug or simultaneous change of at least two backbone drugs) or at a treatment interruption lasting >14 days.

cART was defined as a protease inhibitor (PI)-based, non-nucleoside reverse transcriptase inhibitor (NNRTI), or fusion inhibitor-based regimen, in combination with at least two nucleoside or nucleotide reverse transcriptase inhibitors (NRTIs), or a triple NRTI regimen including abacavir or tenofovir. Virologic response was defined as time to the first of two successive HIV-RNA <500 copies/ml. Virologic failure was time to the first of two HIV-RNA ≥500 copies/ml, to the first (unconfirmed) measurement of ≥1000 HIV-RNA copies/ml, or to 6 months after cART for those not responding by that time.

### Laboratory methods

Sequencing was performed by investigators in the country of origin, using various genotypic resistance assays. HIV-1 subtype was derived from nucleotide sequence data spanning the entire protease gene and at least codons 41–236 of reverse transcriptase using the Rega algorithm [19]. HIV-RNA values were also determined locally. We defined response as HIV-RNA to <500 copies/ml as this was the limit of detection for the least sensitive assay used.

### Statistical analysis

Cumulative incidence of response or failure, in the presence of the competing event of switching to a new cART regimen, was estimated through the non-parametric Aalen-Johansen estimator [20]. The cause-specific hazards of response and failure were analysed using the Cox proportional hazards model [21]. We also examined CD4 count change following cART based on a piece-wise linear mixed model, with change in slope 3 months after initiation. Multivariable models were adjusted for gender, risk group [men having sex with men (MSM), sex between men and women (MSW), injecting drug users (IDU), other/unknown], age at cART initiation, pre-cART antiretroviral experience (naive, experienced), time from seroconversion to cART initiation (<6, 6–48, ≥48 months indicating early, medium and late treated), initial cART (unboosted PI, boosted PI, NNRTIs, other), and log_10_ HIV-RNA levels at cART initiation (baseline).

Monte Carlo based power calculations (500 replications/outcome) were used to determine the minimum effect sizes required to achieve a power of at least 0.80 at an alpha level of 0.05 given the sample size per subtype, follow-up time (simulated by an exponential distribution of censoring times), CD4 cell count distribution over time (simulated by a random effects piecewise linear model) and rates of virologic response/failure (simulated by Weibull and Gompertz distributions, respectively) in our study. Parameters values for the simulation were based on real data analyses. These calculations were performed for all major analyses and for comparisons of subtype B group with the smallest and largest non-B subtype groups.

A set of sensitivity analyses were undertaken. Firstly, to allow for the potentially confounding effect of ethnicity, final models were i) further adjusted for ethnicity (white, black, other, and unknown) or ii) restricted to white only. Secondly, as individuals treated during primary HIV-1 infection may have different responses compared to those treated during chronic infection, final analyses were repeated excluding those who initiated cART within 1 year of seroconversion and with CD4 counts >350 cells/µl. Thirdly, analyses were restricted to those who started cART after year 2000 when boosted PIs became widely available. Fourthly, IDUs were excluded from analyses as response in this group is known to be lower compared to other risk groups (mainly due to poor adherence). Finally, to minimize selection bias, analyses were restricted to those cohorts with >50% of their participants subtyped.

## Results

### Study population characteristics

The CASCADE database, updated in September 2011 within EuroCoord (www.EuroCoord.net), included data from 25,629 individuals of whom 15,175 initiated cART during follow-up. Of these, 8,492 were excluded from all analyses as follows: 156 from the 2 African cohorts, 5,672 who seroconverted <1996, 1 aged <15 years, 1,529 who were on their first cART regimen for <60 days, and 1,134 as CD4 cell count and/or HIV-RNA measurements at cART initiation or while on cART were not available. Of 6,683 eligible individuals, HIV-1 subtype was known for 2,152 (32.2%). Individuals with known subtypes were, on average, more likely to be white, of non sub-Saharan African origin and infected through sex between men. They also had higher viraemia at cART initiation (mean viral load 4.9 vs. 4.5 log_10_ copies/ml; p<0.001); shorter delays between seroconversion and cohort enrolment (median 1 vs. 7 months; p<0.001) and initiated cART at slightly higher CD4 counts (median 330 vs. 316 cells/µl; p<0.001).

Of the 2,152 with known HIV-1 subtype, 54 had HIV-RNA <500 copies/ml at cART initiation and were, therefore, excluded from further analyses. Of the remaining 2,098, the predominant subtypes were B (n = 1706, 81.3%), followed by CRF02_AG (n = 142, 6.8%), A (n = 55, 2.6%), C (n = 53, 2.5%), CRF01_AE (n = 47, 2.2%), other recombinants (n = 47, [2.2%]: 34 with various CRFs and 13 with URFs), and various other subtypes (n = 48 [2.3%]: 14 D, 12 F, 21 G and 1 J). As our main aim was to investigate the effect of each single subtype, we excluded subtypes infecting fewer than 30 individuals. Our analyses were thus restricted to 2003 individuals, the demographic and clinical characteristics of whom are shown in Table 1 according to HIV-1 subtype. Individuals with non-B subtypes were more likely to be black, infected through MSW, slightly older, and more likely to initiate cART in more recent calendar periods (and hence less likely to initiate an unboosted PI-based regimen) compared to those with subtype B. Viraemia and CD4 count at cART initiation were similar across all groups. Individuals with CRF02_AG started cART significantly sooner after seroconversion compared to those infected by other subtypes.

**Table 1:** Demographic and clinical characteristics of the study population at cART initiation.

HIV-1 subtype							
	A (N = 55)	B (N = 1,706)	C (N = 53)	CRF01_AE (N = 47)	CRF02_AG (N = 142)	Overall (N = 2,003)	p-value p-value
Risk group & Gender							<0.001^*^
* MSM* 1	12 (21.8)	1342 (78.7)	12 (22.6)	11 (23.4)	52 (36.6)	1429 (71.3)	
* Male IDU^2^*	1 (1.8)	47 (2.8)	2 (3.8)	1 (2.1)	0 (0.0)	51 (2.5)	
* Female IDU*	0 (0.0)	27 (1.6)	1 (1.9)	0 (0.0)	0 (0.0)	28 (1.4)	
* Male MSW^3^*	18 (32.7)	119 (7.0)	15 (28.3)	26 (55.3)	29 (20.4)	207 (10.3)	
* Female MSW*	22 (40.0)	101 (5.9)	19 (35.8)	9 (19.1)	54 (38.0)	205 (10.2)	
* Other-Unknown*	2 (3.6)	70 (4.1)	4 (7.5)	0 (0.0)	7 (4.9)	83 (4.1)	
Ethnic/racial group							<0.001^*^
* White*	41 (74.5)	1281 (75.1)	41 (77.4)	38 (80.9)	76 (53.5)	1477 (73.7)	
* Black*	7 (12.7)	45 (2.6)	8 (15.1)	2 (4.3)	33 (23.2)	95 (4.7)	
* Other*	1 (1.8)	53 (3.1)	1 (1.9)	4 (8.5)	3 (2.1)	62 (3.1)	
* Unknown*	6 (10.9)	327 (19.2)	3 (5.7)	3 (6.4)	30 (21.1)	369 (18.4)	
Acute infection^4^	25 (45.5)	752 (44.1)	17 (32.1)	19 (40.4)	106 (74.6)	919 (45.9)	<0.001^*^
AIDS before cART	1 (1.8)	76 (4.5)	3 (5.7)	2 (4.3)	3 (2.1)	85 (4.2)	0.586^*^
ART naïve	52 (94.5)	1655 (97.0)	50 (94.3)	47 (100.0)	141 (99.3)	1945 (97.1)	0.158^*^
cART based on							0.027^*^
* Unboosted PI*	6 (10.9)	302 (17.7)	6 (11.3)	4 (8.5)	17 (12.0)	335 (16.7)	
* NNRTI*	21 (38.2)	628 (36.8)	28 (52.8)	22 (46.8)	42 (29.6)	741 (37.0)	
* Boosted PI*	26 (47.3)	703 (41.2)	18 (34.0)	21 (44.7)	77 (54.2)	845 (42.2)	
* 3 class/Other*	2 (3.6)	73 (4.3)	1 (1.9)	0 (0.0)	6 (4.2)	82 (4.1)	
CD4 cell count/mm^3^	329	330	290	309	338	330	0.207^**^
(cells/μl)	(264, 580)	(236, 470)	(213, 393)	(201, 463)	(243, 438)	(235, 468)	
Plasma HIV-RNA (log_10_ c/ml)	4.8 (0.9)	5.0 (0.8)	5.0 (0.9)	4.9 (0.8)	5.0 (0.9)	5.0 (0.8)	0.143^**^
Age (years)	40.6 (13.5)	37.2 (9.3)	38.8 (14.0)	44.1 (11.1)	37.3 (11.0)	37.5 (9.8)	<0.001^**^
Calendar year	2004	2004	2005	2006	2006	2005	<0.001^**^
at cART initiation	(02, 08)	(01, 07)	(02, 07)	(03, 08)	(03, 08)	(01, 08)	
SC to cART (months)	17 (1, 37)	13 (2, 33)	17 (6, 33)	26 (4, 44)	6 (1, 26)	13 (1, 33)	0.001^**^

Numbers in upper panel are N(%) and in lower panel Mean (SD) or Median (IQR).

1 : men having sex with men;^ 2^: intravenous drug users;^ 3^: sex between men and women;^ 4^: as indicated by a short (<30 days) HIV test interval.

*: Chi-square test; ^**^ Kruskal-Wallis test.

Of 607 individuals initiating cART within one year of seroconversion and with CD4 counts >350 cells/µl, 338 (55.7%) were from the PRIMO cohort in France, reflecting the policy in this country during the 1996–2004 period to systematically treat individuals presenting during primary HIV-1 infection. However, the rate of “early” treated subjects did not differ significantly by HIV-1 subtype (p = 0.479). Notably, the majority of those infected with CRF02_AG (93/142, 65.5%) were also from the PRIMO cohort, which may explain the shorter time intervals between seroconversion and cART initiation observed for that subtype.

As the analysis of virological response may be sensitive to the time of HIV-RNA measurements, we compared median follow-up time, number of HIV-RNA measurements and time interval between consecutive measurements across subtypes and found no significant differences (p>0.39 in all cases).

### Virological Response

**Figure 1:**
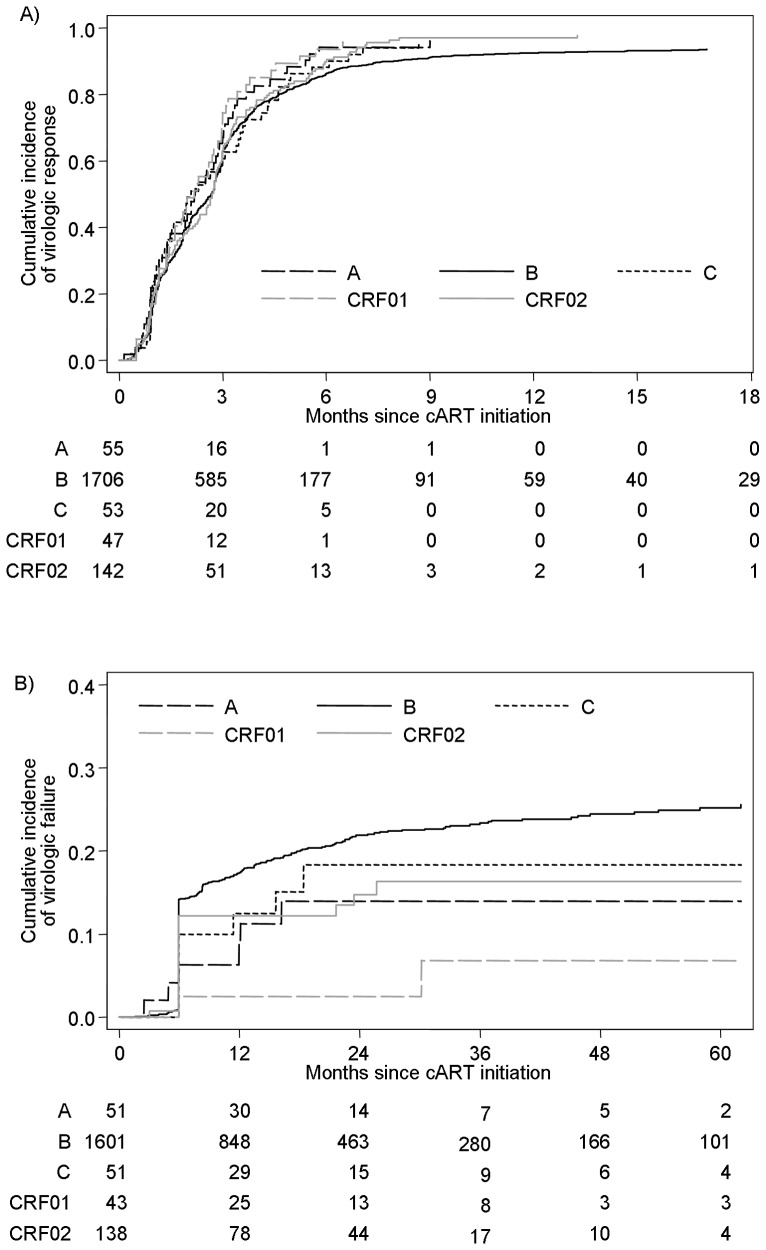
Cumulative incidence of initial virologic response (A) and virologic failure (B) by HIV-1 subtype. Numbers below each subfigure indicate numbers of individuals “at risk” (i.e. subjects not responded (A) or failed (B) and under follow-up).

Of 2003, 1,847 responded virologically with cumulative incidence (95% CI) of 87.0% (85.4, 88.4) at 6 months after cART initiation, which did not differ significantly by HIV-1 subtype (p = 0.097) (Figure 1A).

In an unadjusted analysis, given the low numbers with non-B subtypes and comparing the subtype B group to the smallest (i.e. CRF01_AE) and the largest (i.e. CRF02_AG) non-B subtype groups, we estimated that we would have 80% power, at an alpha level of 0.05, to detect hazard ratios of 1.53 and 1.27, respectively.

Pairwise comparisons through an unadjusted cause-specific proportional hazards model, however, indicated that, compared to those with subtype B, individuals with CRF01_AE and A experienced higher rates of response (HR = 1.37; 95% CI: 1.02–1.84 and 1.28; 0.97–1.70, respectively). Adjusting for factors mentioned in the methods section yielded similar results (Table 2). Rates of response were also higher for individuals with low baseline viraemia, MSM (compared to other groups), those naïve at cART initiation, those initiating cART <6 or ≥48 months after seroconverion and those on boosted PI or NNRTI-based regimens.

**Table 2:** Factors associated with cause-specific hazard of initial virologic response and virologic failure after cART initiation.

	Virologic response			Virologic failure		
Factor	HR	95% C.I.	p-value*	HR	95% C.I.	p-value*
HIV subtype			0.075			0.317
* A*	1.29	(0.96, 1.72)		0.76	(0.33, 1.74)	
* B*	1			1		
* C*	1.20	(0.90, 1.60)		0.71	(0.35, 1.46)	
* CRF01_AE*	1.37	(1.01, 1.86)		0.33	(0.08, 1.33)	
* CRF02_AG*	1.15	(0.96, 1.39)		0.72	(0.45, 1.17)	
Log_10_ HIV-RNA at cART initiation			<0.001			0.029
*per 1 log_10_ c/ml*	0.70	(0.66, 0.75)		1.16	(1.01, 1.32)	
Risk group			<0.001			0.020
* MSM* 1	1			1		
* IDU^2^*	0.64	(0.49, 0.83)		1.84	(1.25, 2.71)	
* MSW^3^*	0.81	(0.71, 0.92)		1.10	(0.82, 1.47)	
* Other-Unknown*	0.95	(0.76, 1.20)		1.19	(0.71, 1.97)	
Pre-cART ART experience			<0.001			<0.001
* Naïve*	1			1		
* Experienced*	0.58	(0.43, 0.79)		2.39	(1.55, 3.68)	
Time from seroconversion to cART initiation			<0.001			
* <6 months*	1					
* [6, 48 months)*	0.71	(0.64, 0.79)				
* ≥48 months*	1.18	(1.02, 1.37)				
cART based on			<0.001			<0.001
* Unboosted PI*	1			1		
* NNRTI*	1.55	(1.34, 1.80)		0.45	(0.35, 0.59)	
* Boosted PI*	1.42	(1.23, 1.64)		0.36	(0.27, 0.48)	
* 3 class/Other*	0.88	(0.68, 1.15)		1.15	(0.79, 1.68)	
Age at cART initiation						0.001
*per 10 years*				0.82	(0.72, 0.92)	

1 : men having sex with men;^ 2^: injecting drug users;^ 3^: sex between men and women. Each factor is adjusted for all other factors in Table.

* global Wald-type tests based on the fit of the corresponding models.

Excluding individuals who initiated cART during the first year of seroconversion with CD4 >350 cells/μl yielded similar results although the difference between A and B was attenuated. Similar results were obtained when IDUs were excluded from the analysis. Restricting analyses to individuals who started cART after year 2000, those belonging to cohorts with >50% of their participants subtyped, and only whites or adjusting also for ethnicity, yielded results compatible with those of the main analysis, although hazard ratios when comparing individuals with non-B subtypes to those with subtype B were slightly attenuated and non-significant (data not shown), most likely due to reduced sample size.

### Virologic failure

After excluding 119 individuals with less than 180 days of available follow up, 158 (8.4%) of the remaining 1,884 failed having initially responded, and 208 (11.0%) had not responded by 6 months. The overall mean (95% CI) estimated cumulative incidence of failure 24 and 48 months after cART initiation was 20.6% (18.7, 22.7) and 23.1% (20.9, 25.3), respectively. There was relatively weak evidence (p = 0.059) to suggest that individuals with non-B subtypes were more likely to experience lower rates of failure (Figure 1B).

Comparing to subtype B, we estimated that the minimum effect sizes (i.e. hazard ratios) that could be detected with 80% power, at an alpha level of 0.05, were 0.42 and 0.59 for subtypes CRF01_AE and CRF02_AG, respectively.

The most pronounced and significant difference was between individuals infected with CRF01_AE and B subtype (unadjusted HR = 0.23, 95% CI: 0.06–0.92; p = 0.039). In multivariable analysis though, differences between subtypes were attenuated with no evidence of a difference in risk of failure rate by subtype (Table 2; overall p = 0.317). Results from the same model indicated that individuals infected through IDU, at younger ages, pre-treated, with higher baseline HIV-1 RNA and with an unboosted PI-based cART, were independently associated with higher probabilities of virological failure.

Sensitivity analyses similar to those performed for the virological response outcome, yielded comparable results with non-B subtypes having mainly protective (i.e. hazard ratios <1) but non-significant effects.

### Immunological response

**Figure 2:**
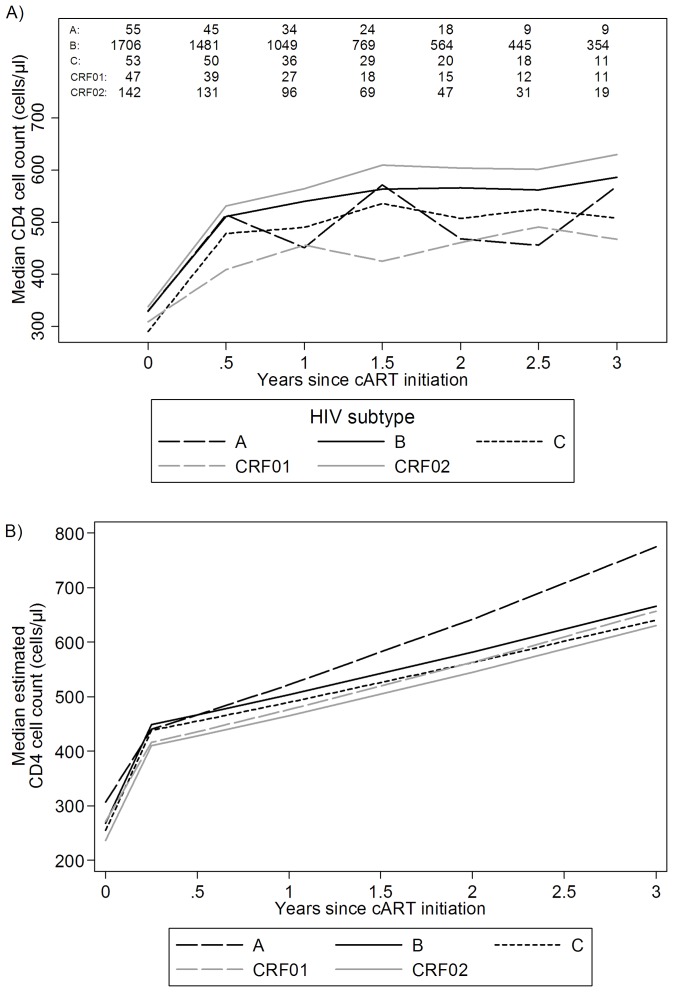
Observed (A) and predicted (B) CD4 cell count by HIV-1 subtype. A: Median profiles (numbers on top indicate individuals contributing measurements at each time point), B: based on a piecewise linear mixed model (Non acute infection, without AIDS at cART initiation, boosted PI cART, SC to cART>4 years, previously naïve, 5 log_10_ c/ml initial viral load, men having sex with men, 30 years old at cART initiation).

Increases in CD4 cell count after cART initiation were biphasic with steep initial (first 3 months) increase followed by milder long-term (after 3 months) increase. Median CD4 cell count profiles by HIV-1 subtype and time since cART initiation are shown in Figure 2A. However, caution is required when interpreting such figures due to their cross-sectional nature as the number of individuals contributing measurements at each time point is not constant and is influenced by missed visits and other censoring mechanisms (mainly switching to a new cART regimen).

Results from an unadjusted piecewise linear mixed model with a knot at 3 months after cART initiation showed that individuals with subtype A experienced slower (p = 0.006) initial and faster (p = 0.014) long-term rates of CD4 increases compared to individuals infected with subtype B. No such differences were detected for any other subtype. After adjusting for all previously mentioned covariates, individuals infected with subtype A still experienced slower initial CD4 increase (p = 0.007) and faster long-term CD4 cell increase (p = 0.012). Estimated initial average increases ranged from 160 to 200 CD4 cells/μl for individuals infected with subtypes A and C, respectively. The estimated increase at two years ranged from 292 to 335 CD4 cells/μl for individuals infected with subtypes CRF01_AE and A, respectively. Estimated median (95% CI) CD4 cell counts at various time points and longitudinal trends by HIV-1 subtype for a typical subgroup are shown in Table 3 and Figure 2B, respectively.

**Table 3:** Estimated median (95% CI) CD4 cell count by HIV-1 subtype and time since cART initiation.

	Time				
HIV subtype	cART initiation	3 months	6 months	1 year	2 years
***A***	307 (261, 357)	440 (380, 505)	467 (406, 532)	522 (458, 590)	642 (561, 728)
***B***	268 (250, 287)	449 (422, 477)	467 (439, 495)	504 (476, 533)	582 (548, 617)
***C***	255 (212, 301)	438 (378, 503)	455 (395, 519)	490 (428, 555)	563 (490, 640)
***CRF01_AE***	271 (225, 321)	416 (355, 483)	436 (374, 502)	476 (412, 545)	563 (484, 648)
***CRF02_AG***	236 (206, 268)	410 (367, 455)	428 (385, 473)	465 (421, 512)	545 (491, 601)

(Non acute infection, without AIDS at cART initiation, boosted PI cART, seroconversion to cART >4 years, previously naïve, ≥5 log_10_ c/ml initial viral load, men having sex with men, 30 years old at cART initiation).

Based on our power calculations, differences in CD4 count gains at 1 year after cART initiation, comparing subtype B group to the smallest (i.e. CRF01_AE) and largest (i.e CRF02_AG) non-B subtype groups of 80 and 50 cells/μL respectively, could be detected with 80% power at an alpha level of 0.05.

Performing the same procedures of sensitivity analyses as mentioned earlier and adjusting for baseline CD4 cell count, yielded results consistent with those of the main analysis.

## Discussion

Using data from CASCADE, a large collaborative study, we had sufficiently large numbers to compare virologic and immunologic response to cART among individuals infected with subtypes A, B, C, CRF01_AE and CRF02_AG. Overall, we found no clinically important differences between different HIV-1 subtypes and virological and immunological response to cART, and virological failure. However, there were some indications that those infected with subtype CRF01_AE and, to a lesser extent, with subtype A, responded sooner compared to subtype B. The difference between CRF01_AE and B subtype persisted in almost all sensitivity analyses. Although all non-B subtypes tended to have lower rates of virological failure compared to subtype B, when comparing each specific non-B subtype to subtype B no significant differences were found. This is an important finding given that cART drugs were generally developed based on efficacy to subtype B whereas the vast majority of the global HIV epidemic comprises non-B subtypes.

Our findings of shorter time to suppression for subtype A, compared to B, and a lack of any difference in failure rates by subtype support those of others [15]. Easterbrook et al [16], reported no significant differences in virological response and subsequent rebound for subtypes B, A, C and CRF02_AG. In that study, they also reported higher rates of virological rebound for subtype D compared with subtype B. In our study we did not have sufficient number of D infections (n = 14) to confirm this finding. On the other hand, recently published data from the Swiss HIV Cohort study (SHCS) [22] have shown that, restricting analyses to whites only, individuals infected with non-B subtypes had a lower risk of virologic failure than those infected with B subtype. In particular, subtypes A and CRF02_AG had improved outcome. In our study, when comparing jointly all non-B subtypes to subtype B the point estimate was the same as the one reported from the SHCS cohort (adjusted HR for non-B/B = 0.68) and statistically significant (p = 0.048). When single subtypes where considered no significant inter-subtype differences were found. This could be due to reduced power, even though the number of individuals infected with CRF02_AG subtype was comparable in the two studies (142 in CASCADE vs. 128 in the Swiss cohort).

Evaluation of virological response may be sensitive to the timing of HIV-RNA measurements. Therefore, differences in HIV-RNA measurements timing by HIV-1 subtype may influence results regarding virologic response. However, in our study we did not find any difference in median follow-up time; number of HIV-RNA measurements or the time interval between consecutive measurements by HIV-1 subtype. Higher baseline viremia is associated with poorer response to cART initiation. In our study baseline viremia was similar across HIV-1 subtypes, which was, in any case, adjusted for in all our analyses.

Most previous studies have not found any significant difference between HIV-1 subtype and rates of CD4 increases after cART initiation [8]–[10], [13], [15], [17], [23]. In one, relatively small, study [14], CD4 increases at 24 months after cART initiation were lower for individuals with non-B subtypes, but this difference was mainly due to the lower baseline CD4 counts for those harboring subtype A. In a larger study [15] lower baseline CD4 in individuals infected with non-B subtypes, compared to those with B subtype, was reported. Rates of CD4 count recovery were similar overall but, due to initial differences, the CD4 count gap between non-B and B infections persisted throughout therapy. In our study, after adjusting for several factors including time from SC to cART initiation, those infected with non-B subtypes, except for subtype A, tended to initiate cART at slightly lower CD4 counts, compared with those infected with B subtype, although the difference was significant only for those infected with CRF02_AG. Those infected with subtype A had lower initial but also faster long-term CD4 increases than those infected with subtype B. When ethnicity was also considered, with or without HIV-1 subtype adjustment, blacks tended to have lower CD4 counts at cART initiation, but rates of CD4 recovery did not vary significantly by ethnicity.

It is known that unboosted PIs have lower efficacy than boosted PI or NNRTI-based regimens. In our study, individuals infected with subtype B, were more likely to initiate unboosted PI-based regimens compared to those with non-B subtypes. This was mainly because they started therapy in earlier calendar periods before the prevalence of non-B subtypes increased in Western cohorts [24]–[29]. As we have controlled for cART regimen, this difference is unlikely to have affected our results. Additionally, a sensitivity analysis restricting data to those who started cART after year 2000 was carried out and results were similar to those of the main analyses in all cases.

Jensen-Fangel et al [23] comparing whites with non-whites in a Danish cohort of HIV-1 seropositives under cART found no significant differences between them in virological response, clinical progression or immunologic response rates. Frater et al [9] found no significant differences in virological response between those infected with B and non-B subtypes (mainly infected with A, C or D). Comparing European with African cohorts, however, while there was no difference in initial (short term) virological response rates, long-term virological response tended to be poorer in African cohorts, indicating that ethnicity, rather than HIV-1 subtype, may be a more important determinant of long-term virologic response to cART [9]. As expected, HIV-1 subtype was strongly associated with ethnicity and risk group in our study. We used a set for sensitivity analyses to allow for potential confounding effects of these factors and, in all cases, results were compatible with those of the main analyses. Although residual confounding can never be ruled out in observational studies, we believe that the lack of any significant and/or clinically important association between HIV-1 subtype and long-term virological response or immune reconstitution rates is unlikely to be attributed to residual confounding. These results are supported by the recent findings from the SHCS that fully controlled for ethnicity [22].

Low adherence is an important determinant of poor virologic response. Adherence data are not collected in CASCADE. Some studies have suggested that adherence levels are lower in immigrants/minorities than in indigenous populations, with this difference being mainly attributed to differences in cultural and socioeconomic factors [30]–[32]. If adherence levels differed by HIV-1 subtype, given that non-B subtypes are more common in non-whites, controlling for adherence would have resulted in even lower rates of virological failure for those infected with non-B subtypes. In the UK CHIC study [15], for example, it was found that those infected with subtype C had increased risk of virologic rebound relative to those infected with subtype B. However, this difference was not observed when virologic rebound, which was likely to be related to non-adherence was excluded.

Although this is one of the largest studies addressing the question of virologic and immunologic response to cART, the numbers of some specific HIV-1 subtypes were relatively low leading to reduced power to detect potential inter-subtype differences. Additionally, we were not able to study the whole spectrum of HIV-1 subtypes due to small numbers. Even larger cohort collaborations are needed to fully address this question across all HIV-1 subtypes. Despite these limitations, our study is one of the few studies that had large enough numbers to compare responses to cART initiation in patients infected with subtypes B, A, C, CRF01_AE and CRF02_AG followed in high-income countries with similar access to care. Although our results are subject to the limitation posed by the observational nature of the study, we have shown: similar or even higher probabilities of virologic response to cART among individuals infected with non-B subtypes compared to those with B subtype, equally low rates of virologic relapse across all subtype groups, and limited subtype effects on immunologic response.

In conclusion, our results suggest that current antiretroviral agents have, at least, similar efficacy when administered to persons infected with non-B subtypes living in high-income countries. This is important and reassuring information to HIV care givers and their patients.

## Supporting Information

Appendix S1 (DOC)
