# Supplementary material for: Virologic and Immunologic Response to cART by HIV-1 Subtype in the CASCADE Collaboration
Source: PLoS One. 2013 Jul 30;8(7):e71174. doi: 10.1371/journal.pone.0071174 (PMC3728088; doi:10.1371/journal.pone.0071174)
Supplement: Appendix S1 — (DOC) [file pone.0071174.s001.doc]

**Appendix**

**CASCADE Steering Committee**: Julia Del Amo (Chair), Laurence Meyer (Vice Chair), Heiner C. Bucher, Geneviève Chêne, Osamah Hamouda, Deenan Pillay, Maria Prins, Magda Rosinska, Caroline Sabin, Giota Touloumi.

**CASCADE Co-ordinating Centre**: Kholoud Porter (Project Leader), Ashley Olson, Kate Coughlin, Sarah Walker, Abdel Babiker.

**CASCADE Clinical Advisory Board:** Heiner C. Bucher, Andrea De Luca, Martin Fisher, Roberto Muga

**CASCADE Collaborators**: **Austria:** Austrian HIV Cohort Study (Robert Zangerle); **Australia** PHAEDRA cohort (Tony Kelleher, David Cooper, Pat Grey, Robert Finlayson, Mark Bloch) Sydney AIDS Prospective Study and Sydney Primary HIV Infection cohort (Tony Kelleher, Tim Ramacciotti, Linda Gelgor, David Cooper, Don Smith); **Canada** South Alberta clinic (John Gill); **Estonia** Tartu Ülikool (Irja Lutsar); **France** ANRS CO3 Aquitaine cohort (Geneviève Chêne, Francois Dabis, Rodolphe Thiebaut, Bernard Masquelier), ANRS CO4 French Hospital Database (Dominique Costagliola, Marguerite Guiguet), Lyon Primary Infection cohort (Philippe Vanhems), French ANRS CO6 PRIMO cohort (Marie-Laure Chaix, Jade Ghosn), ANRS CO2 SEROCO cohort (Laurence Meyer, Faroudy Boufassa); **Germany** German cohort (Osamah Hamouda, Claudia Kücherer, Barbara Bartmeyer); **Greece** AMACS (Paparizos V, Gargalianos-Kakolyris P, Lazanas M); Greek Haemophilia cohort (Giota Touloumi, Nikos Pantazis, Olga Katsarou); **Italy** Italian Seroconversion Study (Giovanni Rezza, Maria Dorrucci), ICONA cohort (Antonella d’Arminio Monforte, Andrea De Luca.) **Netherlands** Amsterdam Cohort Studies among homosexual men and drug users (Maria Prins, Ronald Geskus, Jannie van der Helm, Hanneke Schuitemaker); **Norway** Oslo and Ulleval Hospital cohorts (Mette Sannes, Oddbjorn Brubakk, Anne-Marte Bakken Kran); **Poland** National Institute of Hygiene (Magdalena Rosinska); **Spain** Badalona IDU hospital cohort (Roberto Muga, Jordi Tor), Barcelona IDU Cohort (Patricia Garcia de Olalla, Joan Cayla), CoRIS-scv (Julia del Amo, Santiago Moreno, Susana Monge; Madrid cohort (Julia Del Amo, Jorge del Romero), Valencia IDU cohort (Santiago Pérez-Hoyos); **Switzerland** Swiss HIV Cohort Study (Heiner C. Bucher, Martin Rickenbach, Patrick Francioli); **Ukraine** Perinatal Prevention of AIDS Initiative (Ruslan Malyuta); **United Kingdom** Health Protection Agency (Gary Murphy), Royal Free haemophilia cohort (Caroline Sabin), UK Register of HIV Seroconverters (Kholoud Porter, Anne Johnson, Andrew Phillips, Abdel Babiker), University College London (Deenan Pillay). **African cohorts:** Genital Shedding Study (US: Charles Morrison; Family Health International, Robert Salata, Case Western Reserve University, Uganda: Roy Mugerwa, Makerere University, Zimbabwe: Tsungai Chipato, University of Zimbabwe); International AIDS Vaccine Initiative (IAVI) Early Infections Cohort (Kenya, Rwanda, South Africa, Uganda, Zambia: Pauli N. Amornkul, IAVI, USA; Jill Gilmour, IAVI, UK; Anatoli Kamali, Uganda Virus Research Institute/Medical Research Council Uganda; Etienne Karita, Projet San Francisco, Rwanda).

**EuroCoord Executive Board**: Geneviève Chêne, University Bordeaux Segalen, France; Dominique Costagliola, Institut National de la Santé et de la Recherche Médicale, France Julia Del Amo, Instituto de Salud Carlos III, Spain; Carlo Giaquinto, Fondazione PENTA, Italy; Di Gibb, Medical Research Council, UK; Jesper Grarup, Københavns Universitet, Denmark; Ole Kirk, Københavns Universitet, Denmark; Bruno Ledergerber, University of Zurich, Switzerland; Laurence Meyer, Institut National de la Santé et de la Recherche Médicale, France; Alex Panteleev, St. Petersburg City AIDS Centre, Russian Federation; Andrew Phillips, University College London, UK, Kholoud Porter (Chair), Medical Research Council, UK; Caroline Sabin (Scientific Coordinator), University College London, UK, Claire Thorne, University College London, UK; Stephen Welch, Fondazione PENTA, UK.

**EuroCoord Council of Partners**: Jean-Pierre Aboulker, Institut National de la Santé et de la Recherche Médicale, France; Jan Albert, Karolinska Institute, Sweden; Silvia Asandi , Romanian Angel Appeal Foundation, Romania; Geneviève Chêne, University Bordeaux Segalen, France; Dominique Costagliola, INSERM, France; Antonella d’Arminio Monforte, ICoNA Foundation, Italy; Stéphane De Wit, St. Pierre University Hospital, Belgium; Frank De Wolf (Chair), Stichting HIV Monitoring, Netherlands; Julia Del Amo, Instituto de Salud Carlos III, Spain; José Gatell, Fundació Privada Clínic per a la Recerca Bíomèdica, Spain; Carlo Giaquinto, Fondazione PENTA, Italy; Osamah Hamouda, Robert Koch Institut, Germany; Igor Karpov, University of Minsk, Belarus; Bruno Ledergerber, University of Zurich, Switzerland; Jens Lundgren, Københavns Universitet, Denmark; Ruslan Malyuta, Perinatal Prevention of AIDS Initiative, Ukraine; Claus Møller, Cadpeople A/S, Denmark; Andrew Phillips , University College London, UK; Kholoud Porter, Medical Research Council, United Kingdom; Maria Prins, Academic Medical Centre, Netherlands; Aza Rakhmanova, St. Petersburg City AIDS Centre, Russian Federation; Jürgen Rockstroh, University of Bonn, Germany; Magda Rosinska, National Institute of Public Health, National Institute of Hygiene, Poland; Claire Thorne, University College London, UK; Giota Touloumi, National and Kapodistrian University of Athens, Greece; Alain Volny Anne, European AIDS Treatment Group, France.

**EuroCoord External Advisory Board**: David Cooper, University of New South Wales, Australia; Nikos Dedes, Positive Voice, Greece; Kevin Fenton, Health Protection England, UK; David Pizzuti, Gilead Sciences, USA; Marco Vitoria, World Health Organisation, Switzerland.

**EuroCoord Secretariat:** Kate Coughlin, MRC Clinical Trials Unit, UK; Silvia Faggion, Fondazione PENTA, Italy; Lorraine Fradette, MRC Clinical Trials Unit; Richard Frost, MRC Regional Centre London, UK; Christine Schwimmer, University Bordeaux Segalen, France; Martin Scott, UCL European Research & Development Office, UK.
